# GMFNet: A GADF–Mamba Fusion Network for Soybean Seed Hyperspectral Classification

**DOI:** 10.3390/foods15122188

**Published:** 2026-06-17

**Authors:** Chu Zhang, Kai Gao, Xiaoyu Fu, Wenjie Liu, Qinfeng Zhang, Biyao Jin, Guoyi Yu, Junwei Sun, Shenhui Shen, Lei Zhou, Xiaoping Wu, Hengnian Qi, Lu Huang, Chenchen Xue

**Affiliations:** 1 School of Information Engineering, Huzhou Normal University, Huzhou 313000, China; 2 College of Mechanical and Electronic Engineering, Nanjing Forestry University, Nanjing 210037, China; 3 Institute of Industrial Crops, Jiangsu Academy of Agricultural Sciences, Nanjing 210014, China

**Keywords:** deep learning, seed classification, hyperspectral imaging, GADF, feature fusion

## Abstract

Soybean is an important food and oil crop, and rapid nondestructive identification of seed cultivars is crucial for seed purity inspection, varietal certification, breeding management and food-quality control. However, the global spectral profiles of individual soybean seeds from different cultivars are often highly similar, making it difficult for single-representation models to simultaneously capture spectral sequential dependency and inter-band relational structure. To address this issue, this study proposes a GADF–Mamba Fusion Network (GMFNet) for soybean seed hyperspectral classification. Hyperspectral images of 24,800 seeds from eight cultivars were acquired, and reflectance spectra in the range of 900–1700 nm were collected. After preprocessing, 200 effective bands were retained. The preprocessed one-dimensional spectral sequence was fed into a Mamba-based branch to model continuous wavelength dependency and global spectral evolution, while the same sequence was transformed into a GADF image, resized to 208 × 208, and input into a ResNet18-based structural branch to extract inter-band relational features. The two heterogeneous representations were then integrated through a weighted feature fusion module for final classification. Experimental results showed that Mamba achieved the best test accuracy (0.8721) among the raw spectral models, whereas ResNet18 achieved the best test accuracy (0.8737) among the GADF-based structural models. More importantly, the proposed weighted fusion strategy achieved the best overall performance, reaching validation and test accuracies of 0.9039 and 0.9011, respectively. These results demonstrate that spectral sequential information and GADF-based structural semantics are highly complementary. Overall, the proposed framework provides an effective hyperspectral solution for single-seed soybean cultivar identification and shows potential for non-destructive automated quality control in food-industry applications.

## 1. Introduction

Soybean is a globally significant crop, serving as a major source of high-quality protein and vegetable oil for human consumption, animal feed, and various industrial products. Its seed quality and varietal purity directly affect sowing quality, processing efficiency, and downstream product value [1]. In germplasm identification, breeding selection, commercial circulation, and quality supervision, the establishment of a rapid, non-destructive, and accurate soybean seed recognition method is of considerable practical importance [2]. Conventional approaches for seed cultivar identification, including manual morphological inspection, chemical analysis, and molecular marker-based methods, can provide useful information but differ substantially in practical applicability [2]. Manual inspection is relatively simple and inexpensive, but it strongly depends on operator experience and is unsuitable for distinguishing visually similar cultivars at large scale. Chemical and molecular methods generally provide more specific information, but they usually require sample preparation, reagents, specialized instruments, trained personnel, and longer analysis time; some procedures are also destructive and therefore unsuitable for continuous single-seed screening. In contrast, hyperspectral imaging enables non-destructive acquisition of spatial–spectral information from individual seeds without chemical reagents and is more compatible with automated quality-control workflows. For food-industry applications, improvements in seed cultivar classification accuracy have practical significance beyond model evaluation, because reducing misclassification supports cultivar purity control, raw material consistency, and reliable quality-control decisions in large-scale screening scenarios. Therefore, a more accurate non-destructive identification model is valuable for reducing cultivar admixture risk and supporting standardized seed and food-quality control [3].

Hyperspectral imaging (HSI) extends conventional digital imaging by integrating spatially resolved image acquisition with spectroscopic measurement. Unlike RGB images, which mainly encode external phenotypic information such as color, shape, size, and surface texture, HSI records wavelength-dependent reflectance responses associated with the physicochemical characteristics and internal optical properties of biological samples [4]. This advantage is particularly valuable for crop seed and grain quality inspection, where different cultivars or varieties may exhibit highly similar external morphology but differ subtly in biochemical composition, internal structure, and spectral response [3,5]. In the near-infrared region, informative spectral intervals are commonly associated with overtone and combination vibrations of hydrogen-containing bonds. For example, bands around 1100–1300 nm are often related to C–H overtone absorption and are associated with lipid- and carbohydrate-related constituents, whereas regions around 1400–1500 nm are mainly influenced by O–H absorption related to moisture. In addition, spectral responses in the 1500–1650 nm range are generally linked to N–H and C–H related absorptions, corresponding to protein- and organic constituent-related information. Therefore, near-infrared spectral features in these chemically relevant intervals can provide complementary information to external image-derived traits and offer a more informative basis for distinguishing visually similar crop seeds and grain materials than conventional digital imaging alone. In recent years, with the rapid development of machine learning and deep learning, substantial progress has been achieved in HSI-based seed classification [6], especially in one-dimensional deep models that directly learn from raw spectral sequences [7].

Representative studies have demonstrated the effectiveness of HSI in crop seed and grain analysis. Qiu et al. applied HSI combined with convolutional neural networks to single rice seed variety identification, showing that deep learning models could effectively learn discriminative spectral features from individual seeds [8]. Zhao et al. used near-infrared HSI covering 874–1734 nm for maize seed variety classification, in which 12,900 seeds from three varieties were analyzed and the optimized model achieved a prediction accuracy of 91.00% [9]. Bao et al. further applied HSI combined with chemometric models to wheat grain variety classification and reported a classification accuracy of up to 91.3% [10]. In oat seed identification, Wu et al. demonstrated that HSI integrated with an end-to-end deep convolutional neural network achieved a testing accuracy of 99.19% [11]. Overall, these studies demonstrate the effectiveness of HSI as a non-destructive analytical technology for crop seed and grain classification. By integrating spatial imaging with spectral measurement, HSI provides a more informative basis for high-throughput classification and quality evaluation, especially when phenotypic differences among crop materials are subtle.

Existing HSI-based methods for soybean and related seed classification can generally be divided into three categories. The first category is based on conventional machine learning models, such as support vector machines, random forests, and partial least squares discriminant analysis, usually in combination with handcrafted features and spectral preprocessing [2]. Although these methods are relatively simple, their ability to characterize complex nonlinear patterns and high-dimensional feature couplings remains limited. The second category treats the raw spectrum as a one-dimensional sequence and employs convolutional neural networks, recurrent neural networks, Transformers, and related variants to model local spectral variations and global wavelength dependencies [12,13,14]. These approaches are effective in preserving the sequential semantics of raw spectra, but their modeling focus is still mainly placed on sequential dependency, while the explicit structural relationships among spectral bands remain insufficiently explored. The third category attempts to enhance structural modeling by converting spectra into two-dimensional representations or by introducing multi-branch feature fusion [15]. However, for single-seed soybean hyperspectral data, how to preserve the sequential information of raw spectra while explicitly unfolding inter-band differential relationships and effectively coordinating these two representations remains insufficiently studied.

From the perspective of signal representation, the hyperspectral signature of a single soybean seed is inherently both a continuous wavelength sequence and a carrier of rich inter-band relational structure. The raw one-dimensional spectrum naturally preserves the true wavelength order and its continuous variation pattern, making it suitable for modeling overall spectral evolution and long-range wavelength dependency. In contrast, the Gramian Angular Difference Field (GADF) transforms a one-dimensional spectrum into a two-dimensional relational image, allowing inter-band differential relationships that are implicitly embedded in the sequence to be explicitly expressed [16,17]. Therefore, raw spectral sequential representation and GADF structural representation are not mutually substitutive; instead, they are more likely to provide complementary discriminative information. Meanwhile, with the recent development of sequence modeling methods, Mamba has emerged as a promising backbone for one-dimensional spectral learning due to its favorable balance between long-range dependency modeling and computational efficiency [18,19]. Nevertheless, relying solely on a spectral branch is still insufficient for exploiting latent structural relationships in hyperspectral signals, while relying solely on transformed two-dimensional representations cannot fully replace the sequential semantics carried by the original spectra. Therefore, building a collaborative classification framework that jointly utilizes raw one-dimensional spectra and GADF-based two-dimensional structural representation is of substantial significance for improving soybean seed hyperspectral classification.

To address the above issues, this study constructs a GADF–Mamba Fusion Network (GMFNet) for soybean seed hyperspectral classification. The proposed network consists of a spectral branch and a GADF structural branch. Specifically, the one-dimensional branch takes the spectral sequence as input and employs Mamba to model continuous wavelength dependencies and overall spectral evolution, while the two-dimensional branch takes GADF relational images as input to extract structural patterns of inter-band differential relationships. Subsequently, a weighted feature fusion mechanism is introduced to adaptively integrate these heterogeneous representations at the feature level, thereby enhancing the discrimination of fine-grained soybean seed categories. The main contributions of this work are summarized as follows:

(1) A joint representation framework integrating raw one-dimensional spectral sequences and GADF-based two-dimensional relational images is established to enhance the structural expressiveness of single-seed soybean hyperspectral data.

(2) An asymmetric collaborative network consisting of a spectral branch and a GADF structural branch is constructed to jointly model continuous wavelength dependencies and explicit inter-band relational structures.

(3) A weighted feature-level fusion mechanism is introduced to improve the complementary utilization of spectral features and GADF structural features, thereby further enhancing classification performance and generalization stability.

## 2. Materials and Methods

### 2.1. Sample Preparation

Soybean seeds were used in this study to construct a spectroscopic dataset for varietal classification. The samples were obtained from the corresponding germplasm collection and comprised eight soybean cultivars, namely Rudong Popi Feng, Hanjiang Hongdadou, Liyang Chadou Bayuehuang, Taicang Ziyoudou, Dantu Huangdou, Nantong Yuandou, Yangzhou Wuyuedou, and Wujin Xizi. For each cultivar, 3100 seeds were selected, resulting in a total of 24,800 individual seed samples. During dataset construction, each seed was assigned a unique sample index, and its cultivar identity was checked according to the corresponding cultivar label, sample record, and batch information. The acquired hyperspectral images and the subsequently extracted spectral files were organized under the same sample indices, establishing a one-to-one correspondence among the physical seeds, cultivar labels, image data, and spectral data. After seed-region spectral extraction, the spectra were subjected to wavelength trimming and smoothing. The processed spectral samples were then divided into training, validation, and test sets at a ratio of 4:1:1 using three different random seeds, generating three independent dataset partitions. Within each partition, stratified sampling was performed according to cultivar labels, and each seed was assigned to only one subset to avoid sample overlap among the training, validation, and test sets. Wavelength-wise standardization was subsequently performed within each partition using only the corresponding training-set statistics. The cultivar names, class labels, and sample distributions in the training, validation, and test sets are summarized in Table 1.

Prior to hyperspectral acquisition, all samples were manually inspected, and impurities, broken kernels, damaged seeds, shriveled seeds, moldy seeds, insect-damaged seeds, and other visibly abnormal materials were removed. Only plump, intact, and morphologically normal seeds were retained for subsequent analysis. This procedure was completed before spectral acquisition and model development, and the same screening criteria were applied consistently to all cultivars. No sample was selected according to spectral characteristics, classification results, or model performance. This screening step was used to reduce non-cultivar-related spectral variability caused by surface defects, contamination, fungal infection, mechanical damage, or abnormal moisture status, so that the constructed dataset mainly reflected cultivar-associated spectral differences rather than condition-related artifacts.

Before data collection, seeds from each cultivar were gently mixed and randomly sampled to reduce potential sampling bias. Spectral acquisition was conducted in batches, and identical environmental conditions and instrument settings were maintained throughout all batches to ensure the reproducibility, consistency, and comparability of the collected spectra. After preparation, each individual seed was treated as an independent sample for subsequent spectral extraction and classification modeling.

### 2.2. Spectral Acquisition and Extraction

Spectral acquisition was performed using a laboratory hyperspectral imaging system consisting of a hyperspectral camera, a stable illumination source, a motorized translation stage, and dedicated control software, LUMO Scanner 2020 software (Spectral Imaging Ltd., Oulu, Finland). In this study, an FX17 hyperspectral camera (Spectral Imaging Ltd., Oulu, Finland) was employed. The camera covered the near-infrared spectral range from 900 to 1700 nm, and a total of 224 spectral bands were initially collected for each sample.

During acquisition, soybean seeds were evenly placed on the same low-reflectance background plate to enhance the contrast between seed regions and the surrounding area. The background plate was used only as an imaging aid for subsequent seed-region segmentation and was not included as part of the spectral input. During region-of-interest extraction, background pixels were excluded, and the reflectance spectrum of each seed was calculated only from pixels belonging to the segmented seed region. Therefore, background-related signals were not used as classification features during model training. The use of a uniform low-reflectance background mainly helped reduce segmentation uncertainty and minimize background interference across samples. The frame rate and exposure time were set to 70.00 Hz and 8.26 ms, respectively, while the speed of the translation stage was fixed at 24.7 mm/s to ensure stable line-scan imaging under consistent measurement conditions.

To obtain calibrated reflectance spectra, white and dark reference correction was conducted prior to subsequent analysis in order to compensate for sensor dark current and uneven illumination. The corrected reflectance spectrum was calculated as

(1) $$R\left(\lambda \right)=\frac{S_{raw}\left(\lambda \right)-S_{black}\left(\lambda \right)}{S_{white}\left(\lambda \right)-S_{black}\left(\lambda \right)}$$

where $S_{raw}(\lambda )$ denotes the original spectral signal of the sample at wavelength λ, $S_{white}(\lambda )$ denotes the white reference spectrum acquired from a standard diffuse reflectance panel, $S_{black}(\lambda )$ denotes the dark reference spectrum obtained under lens-covered conditions, and R(λ) denotes the corrected reflectance spectrum.

**Figure 1:**
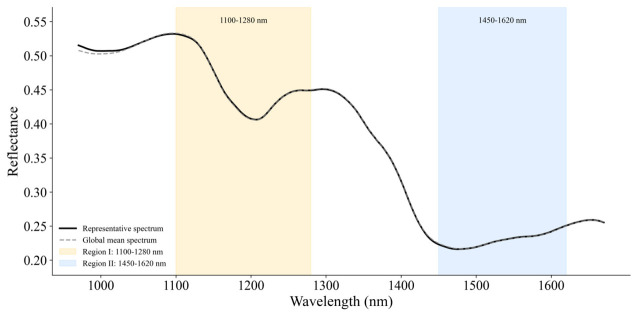
Representative reflectance spectrum and global mean spectrum of soybean seed samples with main spectral response regions.

After reflectance correction, the effective region of each individual soybean seed was identified from the hyperspectral image. Pixel-wise spectra within the seed region were then extracted and averaged to generate a one-dimensional spectral curve for each seed. A representative reflectance spectrum from a single soybean seed sample and the global mean spectrum calculated from all extracted seed spectra are shown in Figure 1 to provide an intuitive example of the spectral characteristics obtained during hyperspectral acquisition and extraction. The representative spectrum closely followed the global mean spectrum, indicating that the selected sample reflected the typical near-infrared optical response pattern of soybean seeds. Overall, the spectrum showed relatively high reflectance in the short-wavelength region, followed by an evident decrease within Region I (1100–1280 nm). This interval is commonly associated with C–H overtone absorption, which is related to organic constituents such as lipids and carbohydrates in soybean seeds. In Region II (1450–1620 nm), the reflectance remained at a relatively low level and then gradually increased with wavelength. This region is generally attributed to O–H and N–H overtone absorption, corresponding to moisture- and protein-related spectral responses. Therefore, Region I and Region II were highlighted as the main spectral response regions involved in the subsequent analysis. Accordingly, each soybean seed corresponded to one independent spectral sample, and the extracted one-dimensional reflectance spectrum was used as the basis for subsequent preprocessing, spectral sequence modeling, and construction of the GADF-based two-dimensional structural representation.

### 2.3. Spectral Preprocessing and Data Preparation

**Figure 2:**
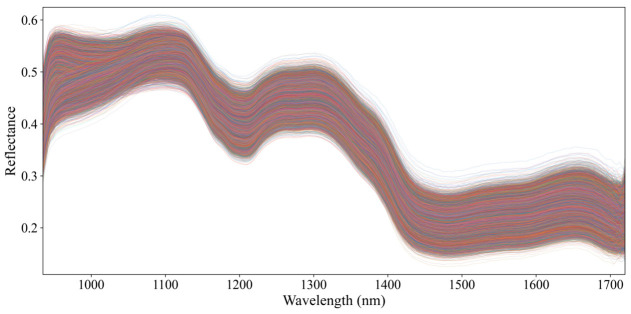
Raw reflectance spectra of soybean seed samples before wavelength trimming and smoothing. The different colors indicate individual spectral curves rather than cultivar labels, and are used only to improve the visual distinguishability of densely overlapping spectra.

Figure 2 shows the raw reflectance spectra of soybean seed samples before wavelength trimming and smoothing. The spectra exhibited generally consistent profiles across samples, indicating that the acquired hyperspectral data captured stable near-infrared response characteristics of soybean seeds. However, relatively large fluctuations were observed at both ends of the wavelength range, especially near the short- and long-wavelength boundaries. These unstable responses were mainly associated with the low signal-to-noise ratio and sensor-related noise in the edge bands. Therefore, to ensure the reliability of the spectral data used for subsequent modeling, the first 10 bands and the last 14 bands were removed, and the remaining 200 bands with relatively stable responses were retained for further analysis.

To suppress high-frequency noise while preserving the overall spectral profile, a five-point moving-average smoothing operation was applied to each retained spectral curve [20]. For each wavelength position, the smoothed reflectance value was calculated as the average of the reflectance values within a centered five-band window. The smoothing window size was determined through a preliminary comparison of different window lengths. A smaller window provided insufficient noise suppression, whereas a larger window tended to over-smooth local spectral variations that may contain cultivar-related information. Therefore, the five-point moving-average window was selected to balance noise reduction and preservation of the main spectral features.

After smoothing, wavelength-wise z-score standardization was further performed to reduce scale differences among spectral variables and improve the numerical stability of model training. For each random-seed data partition, the mean and standard deviation of each wavelength were calculated only from the training samples, and the same standardization parameters were then applied to the validation and test sets to avoid information leakage. When 10-fold cross-validation was used during model training, the standardization parameters were recalculated within each fold using only the corresponding training subset and then applied to the held-out fold. The standardized spectral value was calculated as:

(2) $$x_{i,j}^{\prime }=\frac{x_{i,j}-\mu_{j}}{\sigma_{j}},$$

where $x_{i,j}$ denotes the smoothed reflectance value of the i-th sample at the j-th wavelength, and $\mu_{j}$ and $\sigma_{j}$ denote the mean and standard deviation of the j-th wavelength calculated from the training samples, respectively. After preprocessing, each soybean seed sample was represented by a 200-dimensional one-dimensional spectral vector. The processed spectral vector was used as the input to the spectral sequential branch and as the source signal for constructing the GADF-based two-dimensional structural representation, thereby providing a unified data basis for both one-dimensional sequential modeling and two-dimensional structural modeling in GMFNet.

For dataset construction, three independent random-seed data partitions were generated using a training, validation, and test ratio of 4:1:1. In each partition, stratified sampling was performed according to cultivar labels to maintain the class distribution across the training, validation, and test sets. The detailed sample distribution of each cultivar under the 4:1:1 partitioning strategy is reported in Table 1. Specifically, each individual soybean seed was treated as an independent sample in the modeling process, and its corresponding one-dimensional spectral vector served as the basic input for all compared methods and as the source signal for subsequent GADF transformation, supporting model development, parameter selection, and final performance evaluation. To evaluate the influence of data partitioning on model performance, two additional random-seed data partitions were also generated using the same partition ratio, and the corresponding results were summarized in the subsequent performance analysis.

### 2.4. GMFNet Framework

**Figure 3:**
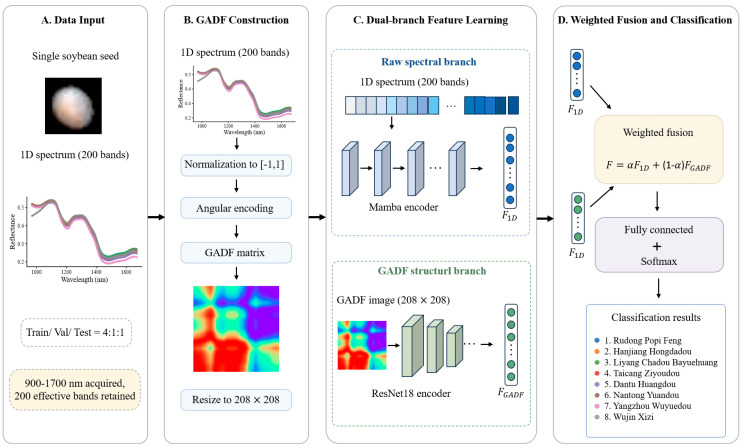
Overall architecture of the proposed GMFNet for soybean seed hyperspectral classification.

To simultaneously exploit the continuous wavelength dependency in single-seed soybean hyperspectral data and the inter-band differential relational structure explicitly unfolded by GADF, a GADF–Mamba Fusion Network (GMFNet) was constructed in this study. As illustrated in Figure 3, the overall pipeline of GMFNet consists of four parts: data input, GADF construction, dual-branch feature learning, and weighted fusion-based classification. For each soybean seed sample, the preprocessed reflectance spectral sequence consisting of 200 effective bands is fed into the network through two parallel pathways. In the first pathway, the spectral sequence is directly input into the spectral branch, where a Mamba encoder is employed to model continuous wavelength dependency, spectral-shape evolution, and long-range inter-band correlations. In the second pathway, the same spectral sequence is transformed into a GADF relational image, resized to 208 × 208 and represented as a single-channel image, and then fed into the GADF structural branch, where a ResNet18 encoder is used to extract two-dimensional structural features characterizing inter-band differential relations. The heterogeneous features produced by the two branches are denoted as $F_{1D}$ and $F_{GADF}$, respectively, and are subsequently integrated through a weighted feature fusion module before being passed to a fully connected classifier and a Softmax layer for final category prediction. Through this design, GMFNet performs joint modeling of spectral sequential semantics and GADF-based relational structural semantics within a unified end-to-end framework, thereby providing a more complete and discriminative representation for soybean seed hyperspectral classification.

#### 2.4.1. GADF-Based Structural Representation Construction

Let the preprocessed spectrum of a soybean seed be denoted as

(3) $$x=\left[x_{1},x_{2},\ldots ,x_{L}\right]\in R^{L},$$

where L=200 denotes the number of retained effective spectral bands. To ensure a valid angular mapping, the spectral vector is first normalized to the interval $\left[-1,\;1\right]$ as

(4) $${\tilde{x}}_{i}=2\cdot \frac{x_{i}-\min\left(x\right)}{\max\left(x\right)-\min\left(x\right)}-1,i=1,2,\ldots ,L.$$

Here, ${\tilde{x}}_{i}$ denotes the normalized spectral response at the i-th band. This step not only unifies the amplitude scale across samples, but also guarantees that all spectral values fall within the valid domain of the inverse cosine function for subsequent angular encoding.

The normalized sequence is then mapped into the angular domain through

(5) $$\phi_{i}=\arccos\left({\tilde{x}}_{i}\right),i=1,2,\ldots ,L.$$

where $\phi_{i}$ denotes the angular encoding corresponding to the spectral response at the i-th band. Through this transformation, spectral amplitudes are converted into angular representations, making it possible to model inter-band relationships in terms of angular differences.

Based on the angular sequence, the GADF matrix is constructed as

(6) $$G_{ij}=\sin\left(\phi_{i}-\phi_{j}\right),i,j=1,2,\ldots ,L.$$

The resulting matrix $G=[G_{ij}]\in R^{L\times L}$ is the original GADF relational representation of the sample, where each element $G_{ij}$ describes the differential relationship between the i-th and j-th spectral bands. Consequently, the entire matrix provides a global relational description of pairwise inter-band differences across the full spectrum. In practical implementation, the original GADF matrix generated from the L-band spectral sequence is further resized to 208×208 and used as a single-channel image for subsequent structural modeling. In this way, the mathematical construction of the inter-band relational matrix is preserved, while the resulting representation is converted into a unified image input suitable for downstream two-dimensional feature learning. Unlike the raw one-dimensional spectrum, which mainly preserves wavelength order and continuous variation, the resized GADF image explicitly unfolds the latent inter-band differential structure into a two-dimensional relational space, enabling the model to learn potentially discriminative structural patterns.

Accordingly, in GMFNet, the same soybean seed is represented in two complementary forms: the raw one-dimensional spectrum for sequential dependency modeling and the GADF relational image for structural relation modeling. This cross-representation construction preserves the sequential semantics of the original spectrum while enhancing the explicit expression of inter-band structural information, thereby providing a unified data basis for subsequent dual-branch collaborative feature learning.

#### 2.4.2. Dual-Branch Feature Learning and Weighted Fusion Mechanism

In GMFNet, the spectral sequential branch and the GADF structural branch are designed to model different aspects of the same sample. The spectral sequential branch takes the preprocessed 200-dimensional spectrum x as input and employs Mamba as the sequential encoder to capture continuous wavelength dependency, global spectral evolution, and potential long-range correlations among distant bands. The feature representation extracted from the spectral branch is formulated as

(7) $$z_{seq}=f_{seq}\left(x\right),z_{seq}\in R^{d},$$

where $f_{seq}(\cdot )$ denotes the Mamba-based one-dimensional sequential encoder, $z_{seq}$ denotes the sequential semantic feature learned from the raw spectrum, and d denotes the feature dimension.

Meanwhile, the GADF structural branch takes the resized single-channel GADF image $G\in R^{1\times 208\times 208}$ as input and employs a two-dimensional structural encoder to extract organizational patterns of inter-band differential relationships in the relational space. In GMFNet, this structural encoder is instantiated as ResNet18. The resulting structural feature representation is written as

(8) $$z_{str}=f_{str}\left(G\right),z_{str}\in R^{d},$$

where $f_{str}(\cdot )$ denotes the two-dimensional structural encoder and $z_{str}$ denotes the structural semantic feature learned from the GADF image. In this way, GMFNet obtains two heterogeneous representations with matched dimensionality from the spectral sequential space and the GADF relational space, respectively.

Since $z_{seq}$ and $z_{str}$ are derived from different representational spaces, they differ in feature granularity and discriminative emphasis. To more effectively integrate these complementary representations, a weighted feature fusion mechanism is introduced at the feature level. Let the fused representation be denoted as $z_{fus}$. It is formulated as

(9) $$z_{fus}=\alpha z_{seq}+\left(1-\alpha \right)z_{str},\alpha \in \left[0,1\right].$$

Here, α denotes the learnable fusion coefficient. Through this mechanism, the model can adaptively regulate the relative contribution of sequential features and structural features during training, thereby improving the collaborative utilization of the two complementary representations.

The fused feature $z_{fus}$ is then fed into the classifier to generate the final category prediction:

(10) $$\hat{y}=Softmax\left(Wz_{fus}+b\right),$$

where $\hat{y}$ denotes the predicted class probability vector, and W and b represent the weight matrix and bias term of the classification layer, respectively.

Overall, GMFNet performs joint modeling of spectral sequential representation and GADF-based structural representation within a unified framework. In this framework, the raw spectrum is processed as a one-dimensional sequential input, while the corresponding GADF is processed as a resized single-channel 208×208 structural image. The sequential branch focuses on continuous wavelength dependency and global spectral evolution, the structural branch focuses on two-dimensional organizational patterns of inter-band differential relationships, and the weighted fusion module integrates these heterogeneous features to improve both discriminative capability and generalization performance for single-seed soybean hyperspectral classification.

### 2.5. Comparative Models

#### 2.5.1. Models Based on Spectral Representation

To systematically evaluate the discriminative capability of spectral sequences for soybean seed classification, SVC, LSTM, 1D-CNN, Transformer, Mamba, CNN-LSTM, and CNN-Transformer were selected as comparative models based on spectral representation. These models span conventional machine learning, recurrent modeling, convolutional modeling, attention-based sequence modeling, state space modeling, and hybrid architectures, thereby providing a comprehensive benchmark for assessing the suitability of different one-dimensional modeling strategies for single-seed soybean hyperspectral classification. For all deep-learning models in this group, the input was the preprocessed reflectance spectral sequence $x\in R^{L}$, where L=200 denotes the number of effective spectral bands, and the final classification layer consistently produced predictions over eight soybean seed categories.

SVC was used as a conventional machine learning baseline to evaluate the separability of the spectral features in a non-deep learning setting. It directly took the reflectance spectral vector consisting of 200 effective bands as input and employed a radial basis function (RBF) kernel for nonlinear classification, with the penalty coefficient set to C=10,000, the kernel parameter set to γ=0.01, and the kernel type set to RBF [21].

LSTM was introduced to model the sequential dependency of spectral responses along the wavelength axis. Each spectrum was treated as a univariate sequence of length 200, with the input dimension set to 1, the hidden dimension set to 128, and the number of recurrent layers set to 2 [22].

1D-CNN was employed to capture local spectral patterns and multi-scale wavelength characteristics through convolutional operators. It adopted a three-layer one-dimensional convolutional architecture with channel numbers of 32, 64, and 128 and kernel sizes of 7, 5, and 3, respectively; each convolutional layer was followed by batch normalization, ReLU activation, and pooling, and the extracted features were aggregated by global average pooling and a fully connected layer for 8-class classification [23].

Transformer was selected to model global wavelength dependencies through self-attention. The input spectrum was first projected into a feature space of dimension d=128, followed by positional encoding to preserve wavelength-order information; the encoder depth was set to 4, the number of attention heads was set to 4, and the hidden dimension of the feed-forward network was set to 256 [24].

Mamba was used as a representative state space model to capture continuous wavelength dependencies and long-range spectral correlations in the spectral sequence. The input sequence was first projected into a 128-dimensional feature space and then processed by 4 stacked Mamba blocks before classification [25].

CNN-LSTM was constructed to combine local spectral pattern extraction and sequential dependency modeling within a unified framework. In this model, two one-dimensional convolutional layers with channel numbers of 32 and 64 and kernel sizes of 7 and 5, respectively, were first employed for local feature extraction, followed by a two-layer LSTM with a hidden dimension of 128 for sequential modeling [26].

CNN-Transformer was further included to investigate the collaborative effect of a convolutional front-end and an attention-based sequence encoder. Its convolutional front-end shared the same parameter configuration as CNN-LSTM, and the extracted features were subsequently fed into a two-layer Transformer encoder with a feature dimension of 128, 4 attention heads, and a feed-forward hidden dimension of 256; in this model, dropout was set to 0.1 to improve training stability [27].

These parameter settings were selected to match the relatively short spectral sequence composed of 200 effective bands while maintaining sufficient representational capacity, thereby providing stable and representative raw-spectral baselines for subsequent comparison with the proposed GMFNet.

#### 2.5.2. Models Based on GADF Structural Representation

To evaluate the effectiveness of GADF structural representation for soybean seed classification, 2D-CNN, ResNet18, Swin-Tiny, Visual Mamba, FasterViT, and ConvNeXtV2 were selected as comparative models based on GADF representation. These models cover different two-dimensional modeling paradigms, including conventional convolutional networks, residual networks, vision Transformers, visual state space models, and modern efficient visual architectures, thereby providing a comprehensive benchmark for assessing the suitability of different structural feature extraction strategies on GADF relational images. For all models in this group, the input was the single-channel GADF-based two-dimensional relational image $G\in R^{1\times 208\times 208}$, and the final classification layer consistently produced predictions over eight soybean seed categories.

2D-CNN was adopted as a basic convolutional structural baseline to extract local relational patterns and hierarchical spatial features from GADF images. It consisted of three two-dimensional convolutional layers with 32, 64, and 128 channels, respectively; all convolutional layers used a kernel size of 3×3, and each layer was followed by batch normalization, ReLU activation, and 2×2 max-pooling. The extracted features were aggregated by global average pooling and a fully connected layer for 8-class classification [28].

ResNet18 was employed to progressively learn hierarchical structural information from the GADF image through residual connections. The standard 18-layer residual architecture was adopted, with the first convolution modified to accept single-channel input and the final fully connected layer adapted to 8 output classes [29].

Swin-Tiny was introduced to capture both local and cross-region structural dependencies in the GADF image through window-based self-attention. It adopted the Tiny configuration, with a patch size of 4, a window size of 7, an embedding dimension of 96, stage depths of $\left[2,2,6,2\right]$, and attention head numbers of $\left[3,6,12,24\right]$; the input embedding layer was modified to accommodate a single-channel 208 × 208 input, and the final classification head was adjusted to 8 classes [30].

Visual Mamba was selected to model long-range structural dependencies in the two-dimensional relational space using a visual state space mechanism. In this study, the input image was partitioned into 4×4 patches and projected into a 96-dimensional feature space, followed by multiple stages of visual state space blocks, and the final classifier was adapted to 8 classes [31].

FasterViT was included as an efficient visual backbone to jointly model local relational patterns and global structural information from GADF images. A lightweight FasterViT configuration was adopted, with the input layer modified for single-channel 208 × 208 GADF images and the final classification head adjusted to produce 8 output categories [32].

ConvNeXtV2 was used as a modern convolutional structural baseline to further assess the effectiveness of advanced visual backbones on GADF images. In this study, the Nano configuration was adopted, with the input image size set to 208 × 208, the input channel number set to 1, the batch size set to 8, the optimizer set to AdamW, the initial learning rate set to $1\times 10^{-4}$, the weight decay set to $1\times 10^{-4}$, and the maximum number of training epochs set to 300; the final classification layer was modified to produce 8 output classes [33].

These parameter settings were adapted to the data format used in this study, namely single-channel 208 × 208 GADF input and eight-class output, so as to maintain sufficient representational capacity while ensuring a fair comparison among different structural models under the same data condition.

### 2.6. Experimental Settings and Evaluation Metric

The proposed GMFNet was implemented in PyTorch 1.13.0 + cu117 and trained in a server environment, where a single NVIDIA RTX 4090 GPU was used for the experiments. In this framework, each soybean seed sample was fed into the network in two coordinated forms: the preprocessed reflectance spectral sequence consisting of 200 effective bands $x\in R^{L}$ was used as the input to the spectral sequential branch, while the corresponding GADF-based two-dimensional relational image $G\in R^{L\times L}$, constructed from the same spectral sequence, was used as the input to the structural branch. The entire network was jointly optimized in an end-to-end manner under a unified classification objective. During training, the AdamW optimizer was adopted with an initial learning rate of 1 × 10^−4^, a batch size of 16, and a maximum of 300 epochs. After training, the model parameters corresponding to the highest validation accuracy were retained for final evaluation on the test set.

In this study, Accuracy, Recall, and F1-score were used to evaluate the classification performance of different models for soybean seed identification. Accuracy was adopted as the primary metric for overall performance comparison, while Recall and F1-score were additionally reported to provide a more comprehensive assessment of test-set recognition performance, particularly in terms of class-level sensitivity and the balance between correct identification and misclassification. Let N denote the total number of samples in the evaluated set, $c_{i}$ denote the ground-truth class label of the i-th sample, and ${\hat{y}}_{i}$ denote the predicted class-probability vector output by the model. The final predicted class is defined as ${\hat{c}}_{i}=arg\;max({\hat{y}}_{i})$. Based on these notations, the classification accuracy is calculated as

(11) $$ACC=\frac{1}{N}\sum_{i=1}^{N}1({\hat{c}}_{i}=c_{i}),\;$$

where 1(⋅) is the indicator function, which equals 1 when the predicted class ${\hat{c}}_{i}$ matches the ground-truth class $c_{i}$, and 0 otherwise. Recall and F1-score were calculated using their standard definitions based on true positives, false positives, and false negatives, and were reported on the test set together with test accuracy. In addition, the classification error was calculated as 1 − test accuracy to quantify the proportion of incorrectly classified samples. For experiments involving different random-seed data partitions, the corresponding metrics were summarized as mean ± standard deviation.

## 3. Results

### 3.1. Spectral Characterization

**Figure 4:**
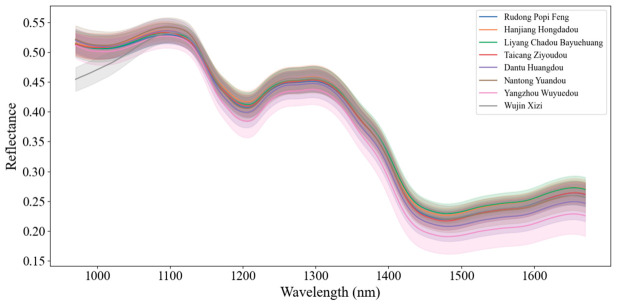
Average reflectance spectral curves of the eight soybean cultivars.

Figure 4 presents the average reflectance spectral curves of the eight soybean cultivars in the near-infrared range, together with their corresponding variation ranges. In terms of the overall trend, the mean spectral curves of different cultivars exhibit a high degree of consistency, indicating that single soybean seeds share similar macroscopic near-infrared response patterns. Such global overlap suggests that the cultivars possess certain common spectral characteristics associated with their major chemical constituents and corresponding absorption behaviors, while also indicating that soybean seed varietal identification is essentially a fine-grained classification task. In other words, the discriminative information is not mainly reflected in large reflectance offsets across the full spectrum, but rather in subtle local variations within specific wavelength intervals.

From the perspective of local spectral morphology, all cultivars remain relatively close in the interval of approximately 980–1130 nm, with a gradual increase around 1100 nm. A noticeable local decline and transition can then be observed within 1130–1230 nm, followed by a relatively smooth plateau-like structure around 1250–1330 nm. As the wavelength further increases, all curves show a pronounced decrease in the 1350–1450 nm interval and reach a low-reflectance valley near 1450 nm, after which the spectra gradually stabilize and exhibit a slight recovery over 1500–1660 nm. These spectral variations are commonly associated with overtone and combination absorptions of hydrogen-containing functional groups in the near-infrared region, especially the vibrational responses of O–H, C–H, and N–H bonds [34]. Considering the compositional characteristics of soybean seeds, such local absorptions and transition regions are generally related to spectral responses from moisture, lipids, proteins, and carbohydrate-associated constituents. Therefore, even when the global spectral profiles are highly similar, subtle yet stable inter-cultivar differences may still arise from differences in chemical composition and microstructural organization.

**Figure 5:**
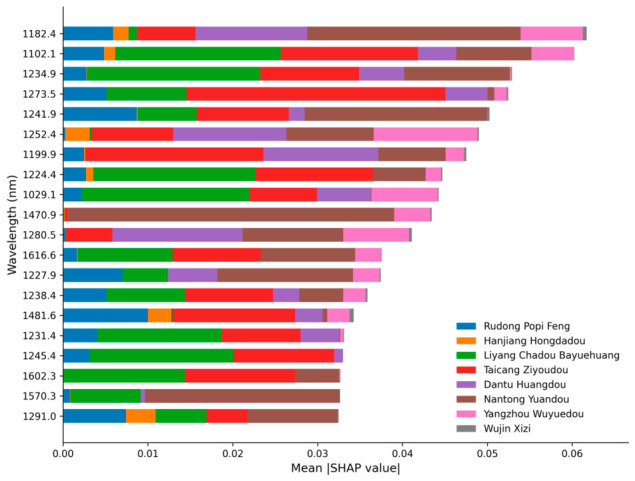
SHAP-based wavelength contribution analysis for soybean cultivar classification.

Although the global spectral profiles were highly similar among cultivars, slight inter-cultivar differences could still be observed within several local spectral regions. To quantitatively characterize these cultivar-dependent spectral changes, SHAP-based wavelength contribution analysis was further conducted. SHAP is a model interpretability method derived from cooperative game theory that assigns an importance value to each input variable according to its contribution to the model prediction. In this study, the mean absolute SHAP value was used to quantify the overall contribution of each wavelength to soybean cultivar discrimination, and wavelengths with larger values were considered to have a stronger influence on the classification decision. The corresponding results are shown in Figure 5. The wavelengths with higher contributions were mainly distributed in the 1100–1290 nm region, including representative wavelengths such as 1188.4, 1102.1, 1234.9, 1273.5, 1241.9, 1252.4, 1199.9, and 1224.4 nm. Additional high-contribution wavelengths were observed around 1470–1620 nm, such as 1470.9, 1570.3, 1602.3, and 1616.0 nm. These regions were consistent with the local spectral transitions observed in Figure 4, demonstrating that cultivar-related differences were mainly expressed as wavelength-level local variations rather than large shifts in the overall spectral shape. The SHAP results further demonstrate that the most influential wavelength regions were closely associated with C–H, O–H, and N–H absorption features, indicating that chemically relevant local spectral variations played an important role in the model decision process. This quantitative interpretation extends the average spectral curve analysis by identifying the specific wavelength regions that carried the most discriminative information for soybean cultivar classification.

**Figure 6:**
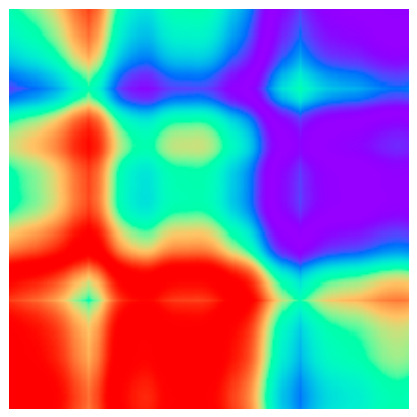
Representative GADF-based two-dimensional relational image of a soybean seed sample.

To further explicitly characterize the inter-band relational structure implicitly embedded in the raw spectra, the GADF transformation was performed based on the spectral sequence analysis. Figure 6 shows the GADF image of a representative sample. It can be observed that local variation patterns in the original one-dimensional spectrum are reorganized into a more intuitive structural distribution in the two-dimensional relational space, where different regions exhibit clearly differentiated color and texture patterns. This suggests that the GADF representation is capable of transforming the latent inter-band differential relationships in the raw spectrum into explicit two-dimensional structural information, thereby providing a more discriminative input basis for subsequent feature extraction in the structural branch.

### 3.2. Classification Performance of Spectral Models

Table 2 summarizes the classification results of the comparative models based on spectral representation on the training, validation, and test sets. All models were trained and evaluated using the 200 effective spectral bands obtained after the preprocessing procedure described in Section 2.3. In addition to accuracy, Recall and F1-score were included to provide a more comprehensive evaluation of test-set classification performance. Overall, the preprocessed spectral sequence exhibited clear discriminative capability for soybean cultivar identification, whereas noticeable performance differences were still observed among different modeling strategies. Among them, Mamba achieved the highest validation and test accuracies of 0.8808 and 0.8721, respectively, and also obtained the highest Recall and F1-score of 0.8719 and 0.8748. These results indicate that Mamba provided the best overall discriminative performance for spectral sequence modeling. In contrast, CNN-LSTM obtained the highest training accuracy of 0.9080, but its validation accuracy, test accuracy, Recall, and F1-score were 0.8628, 0.8662, 0.8660, and 0.8671, respectively, all lower than those of Mamba. This suggests that although the combination of a convolutional front-end and recurrent units enhanced the fitting ability during training, its generalization and test-set classification performance remained slightly inferior to those of the Mamba-based spectral model.

From the perspective of different model categories, the conventional machine learning baseline SVC achieved a test accuracy, Recall, and F1-score of 0.8209, 0.8207, and 0.8188, respectively, indicating that the spectral data themselves already contained a certain degree of class separability, although the capability of SVC to characterize complex nonlinear inter-band relationships remained limited. The recurrent model LSTM yielded a test accuracy of 0.8215, with a Recall of 0.8212 and an F1-score of 0.8240, showing performance close to that of SVC and suggesting that sequential recursive modeling alone was insufficient to fully capture the fine-grained discriminative cues embedded in soybean seed spectra. The convolutional model 1D-CNN improved the test accuracy, Recall, and F1-score to 0.8570, 0.8568, and 0.8586, respectively, implying that local spectral patterns and short-range relationships among neighboring bands played an important role in soybean cultivar identification. The Transformer achieved a test accuracy of 0.8491, with a Recall of 0.8489 and an F1-score of 0.8469, slightly lower than those of 1D-CNN, suggesting that self-attention alone did not show a clear advantage under the current data scale and spectral length. In addition, CNN-Transformer reached a test accuracy, Recall, and F1-score of 0.8210, 0.8208, and 0.8227, respectively, close to those of LSTM and SVC, indicating that a simple serial combination of a convolutional front-end and an attention module did not provide an effective performance gain.

Considering both data-partition variability and training reliability, two additional random-seed data partitions were introduced, and model training was conducted using a 10-fold cross-validation strategy under each partition. The results from the three random-seed data partitions were summarized as mean ± standard deviation in Table 3. Mamba consistently achieved the best overall results among the spectral models, with a mean test accuracy, Recall, and F1-score of 0.8732 ± 0.0019, 0.8730 ± 0.0019, and 0.8737 ± 0.0019, respectively. Classification error was calculated as 1 − test accuracy to quantify the proportion of incorrectly classified samples. Based on the mean test accuracy values, Mamba yielded the lowest classification error of 0.1268, whereas SVC, LSTM, 1D-CNN, Transformer, CNN-LSTM, and CNN-Transformer showed classification errors of 0.1795, 0.1774, 0.1423, 0.1514, 0.1333, and 0.1781, respectively. These results demonstrate that Mamba not only achieved the highest test accuracy but also maintained the lowest misclassification rate across different data partitions. Taken together, different spectral models were able to extract useful discriminative information from soybean seed hyperspectral data, but their performance was strongly influenced by the underlying modeling mechanism. Compared with conventional machine learning models, recurrent networks, convolutional networks, and standard attention-based architectures, Mamba was more suitable for jointly modeling continuous wavelength dependency, global spectral evolution, and long-range inter-band correlations. These results further demonstrate that the preprocessed spectral sequence provides a reliable sequential feature basis for soybean seed classification and subsequent fusion with the GADF-based structural representation.

### 3.3. Classification Performance of GADF-Based Structural Models and Fusion Strategies

Table 4 summarizes the classification results of the GADF-based structural models and fusion strategies on the training, validation, and test sets. In addition to accuracy, Recall and F1-score were included to provide a more comprehensive evaluation of test-set classification performance. Overall, the GADF-based structural representation provided effective two-dimensional discriminative information for soybean seed classification, and all compared visual backbones achieved reasonably stable performance. Nevertheless, clear differences in generalization capability were still observed among different structural models. Among the individual GADF-based models, ResNet18 achieved the highest validation and test accuracies of 0.8668 and 0.8737, respectively, and also obtained the highest Recall and F1-score of 0.8735 and 0.8730. In comparison, the test accuracies of 2D-CNN, Swin-Tiny, Visual Mamba, FasterViT, and ConvNeXt V2 were 0.8498, 0.8505, 0.8500, 0.8655, and 0.8556, respectively. These results indicate that although different two-dimensional models were able to extract discriminative structural information from GADF images, their effectiveness was still strongly influenced by the underlying feature extraction mechanism.

A closer comparison shows that several more complex visual backbones achieved higher training accuracies. For example, Swin-Tiny, Visual Mamba, and ConvNeXt V2 reached training accuracies of 0.9118, 0.9102, and 0.9238, respectively; however, their validation and test performances did not surpass those of ResNet18. This suggests that stronger fitting ability on the training set did not necessarily yield better out-of-sample performance under the current GADF representation and data scale. In contrast, ResNet18 exhibited a more balanced performance across the training, validation, and test sets, indicating that it was more stable in extracting hierarchical structural features from GADF images generated from the preprocessed spectral data. Therefore, ResNet18 was selected as the optimal two-dimensional backbone for the GADF structural branch in the subsequent fusion experiments.

With respect to fusion performance, contact fusion achieved validation and test accuracies of 0.8831 and 0.8895, respectively, outperforming all individual GADF-based structural models. More importantly, Weighted fusion delivered the best overall performance, reaching 0.9618 on the training set, 0.9039 on the validation set, and 0.9011 on the test set. It also achieved the highest Recall and F1-score of 0.9009 and 0.9004, respectively. Compared with the best individual GADF-based structural model, namely ResNet18, Weighted fusion improved the validation and test accuracies by 0.0371 and 0.0274, respectively. Compared with Contact fusion, it further improved the validation and test accuracies by 0.0208 and 0.0116, respectively. These results demonstrate that the spectral sequential representation and GADF-based structural representation are highly complementary, and that the weighted fusion strategy is more effective in coordinating the relative contributions of the two heterogeneous feature types.

Using the GADF datasets generated under the same three random-seed data partitions, model training was also conducted with a 10-fold cross-validation strategy, and the stability of GADF-based structural models and fusion strategies was further evaluated. The corresponding results were summarized as mean ± standard deviation in Table 5. Weighted fusion consistently achieved the best overall performance, with a mean test accuracy, Recall, and F1-score of 0.9014 ± 0.0025, 0.9012 ± 0.0025, and 0.9008 ± 0.0024, respectively. Based on the mean test accuracy values, Weighted fusion yielded the lowest classification error of 0.0986, whereas Contact fusion and ResNet18 showed classification errors of 0.1105 and 0.1262, respectively. These results further confirm that the performance improvement achieved by Weighted fusion was not limited to a single data partition but remained stable across different GADF datasets. Taken together, although a single GADF-based structural representation already provided substantial discriminative capability, the collaborative integration of preprocessed spectral sequential information and GADF-based structural information further released the classification potential of soybean seed hyperspectral data.

### 3.4. Visualization

To further analyze the discriminative capability of the fusion model from the perspective of feature-space distribution, t-distributed stochastic neighbor embedding (t-SNE) was employed to visualize the high-dimensional features of test samples in a two-dimensional space. t-SNE is a widely used nonlinear dimensionality reduction method whose core idea is to map sample similarities in the high-dimensional feature space into a low-dimensional embedding space while preserving local neighborhood structure as much as possible. Specifically, t-SNE first constructs a probability distribution in the high-dimensional space based on pairwise sample distances to describe neighborhood relationships, and then builds a corresponding probability distribution in the low-dimensional space. By minimizing the Kullback–Leibler divergence between the two distributions, samples that are close to each other in the original feature space are encouraged to remain close in the low-dimensional embedding. Therefore, t-SNE is particularly suitable for revealing cluster structure and class separability in learned feature representations.

**Figure 7:**
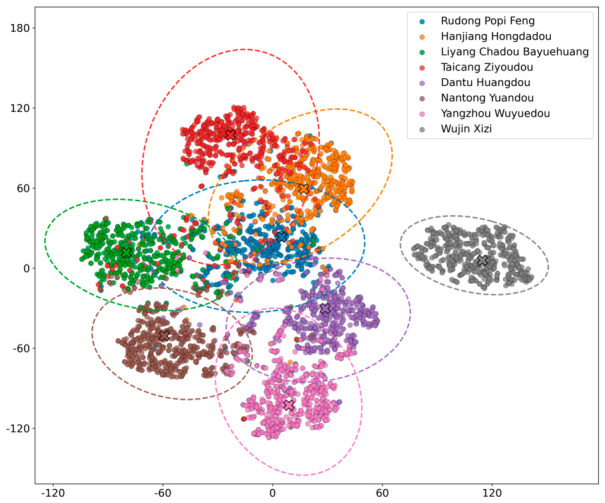
t-SNE visualization of the fused feature space learned by GMFNet on the test set. Dashed ellipses indicate the 95% confidence regions of different soybean cultivar clusters. The cross represents the center of each category.

In this study, the input features used for t-SNE visualization were the final fused representations of the proposed model on the test set, namely the high-dimensional feature vectors before the classifier. The resulting two-dimensional embedding is shown in Figure 7, where 95% confidence ellipses were used to delineate the dispersion regions of different soybean cultivar clusters. Overall, different soybean cultivars formed relatively clear clusters in the embedded space. Most categories exhibited compact intra-class distributions and good inter-class separability, indicating that the fused features learned by GMFNet retained strong discriminative information. The 95% confidence ellipses delineated the dispersion regions of different cultivar clusters and showed that several cultivars occupied relatively independent regions with limited overlap, suggesting clear cluster boundaries and strong category separability in the fused feature space.

At the same time, partial overlap among several neighboring confidence ellipses was still observed, indicating that some soybean cultivars may share similar spectral–structural characteristics and therefore remain more difficult to distinguish. This local overlap provides a visual explanation for the limited misclassifications observed in the normalized confusion matrix analysis and is consistent with the fine-grained nature of soybean seed classification, where different cultivars may exhibit similar global spectral profiles and local structural patterns. Nevertheless, the overall feature distribution remained well organized, demonstrating that the integration of preprocessed spectral sequential information and GADF-based structural information effectively enhanced inter-class separability and intra-class consistency in the feature space.

**Figure 8:**
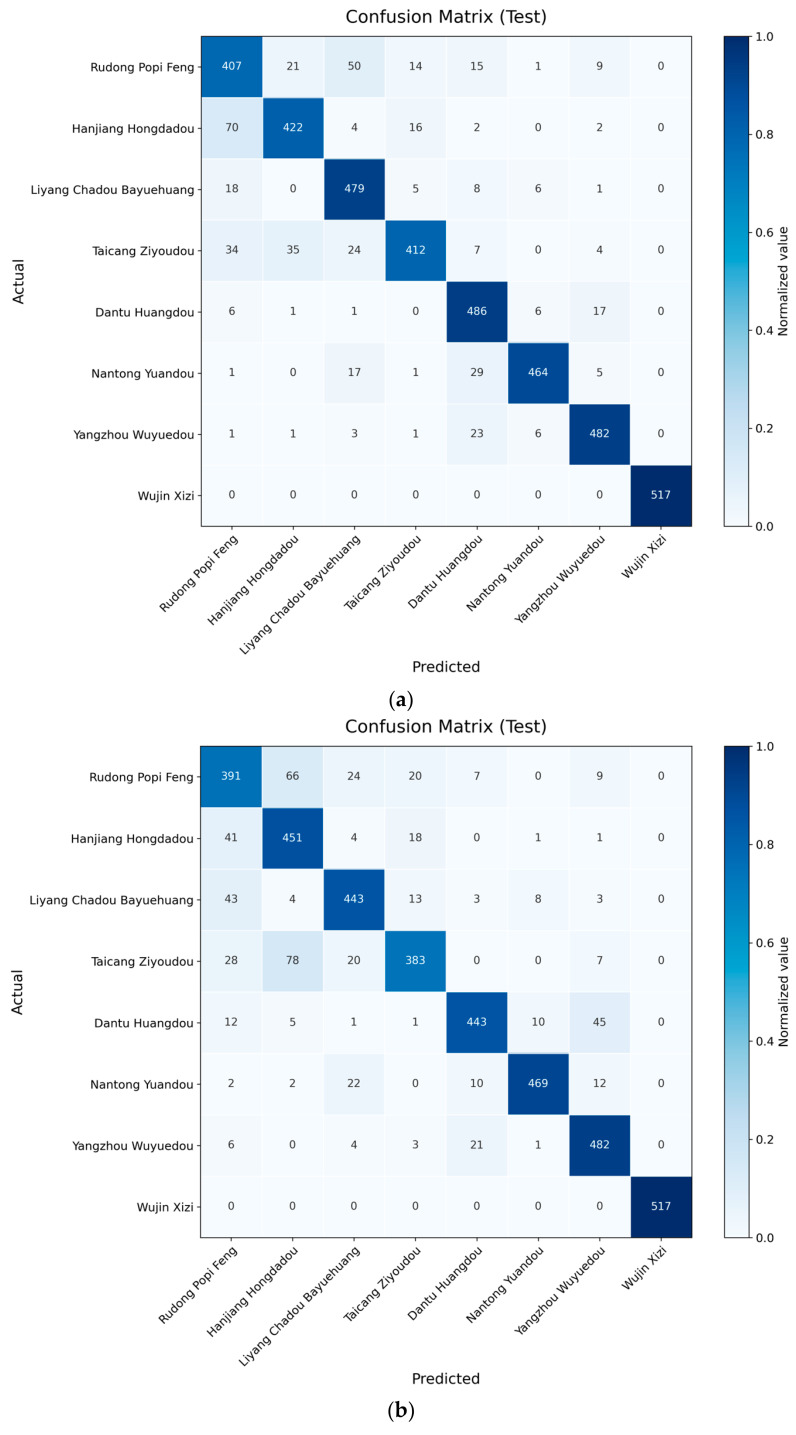

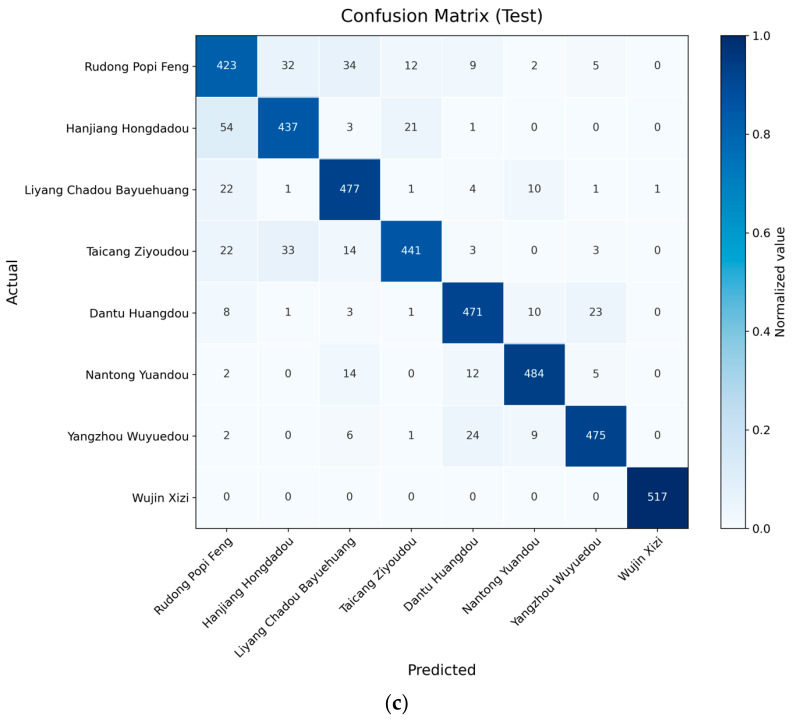
Normalized confusion matrices of representative models on the test set: (**a**) Mamba, (**b**) ResNet18, and (**c**) the proposed GMFNet.

To further evaluate category-level recognition performance, Figure 8 presents the normalized confusion matrices of three representative models on the test set, including Mamba, ResNet18, and the proposed GMFNet. These models correspond to the best-performing spectral sequence model, the best-performing GADF-based structural model, and the final fusion model, respectively. Overall, all three models showed dominant diagonal distributions, indicating that most soybean seed samples were correctly assigned to their corresponding cultivars.

Among the three models, GMFNet exhibited the most consistent and balanced class-wise recognition performance. According to the diagonal responses of the normalized confusion matrix, the class-wise recognition rates of GMFNet ranged from 81.8% to 100.0%, with six of the eight cultivars achieving recognition rates above 85%. In particular, Liyang Chadou Bayuehuang, Dantu Huangdou, Nantong Yuandou, Yangzhou Wuyuedou, and Wujin Xizi showed high recognition rates of 92.3%, 91.1%, 93.6%, 91.9%, and 100.0%, respectively. These results indicate that the proposed fusion framework maintained strong discriminative capability across most soybean cultivars.

Compared with Mamba and ResNet18, GMFNet showed a clearer diagonal pattern and weaker off-diagonal responses, suggesting that the integration of spectral sequential information and GADF-based structural information effectively reduced inter-class confusion. Nevertheless, several off-diagonal responses remained, mainly involving Rudong Popi Feng, Hanjiang Hongdadou, and Taicang Ziyoudou, implying that these cultivars may share similar spectral or structural characteristics. This observation is consistent with the quantitative results in Table 2, Table 3, Table 4 and Table 5, where GMFNet achieved the best overall test performance. Therefore, the normalized confusion matrix analysis further confirms that the proposed fusion strategy improves category-level discriminative ability and enhances the reliability of soybean cultivar identification.

## 4. Discussion

Soybean cultivar classification is essentially a fine-grained recognition problem, in which the difficulty lies not in large reflectance offsets across the full spectrum, but in whether weak perturbations within multiple local wavelength intervals, inter-band coordinated variations, and subtle changes in overall spectral evolution can be effectively captured. Zhu et al. used hyperspectral imaging coupled with convolutional neural networks to identify soybean varieties and demonstrated the effectiveness of deep learning for soybean seed hyperspectral analysis [35]; however, their framework was still mainly organized around image-level convolutional feature learning rather than dedicated modeling of the continuous wavelength dependency embedded in raw one-dimensional spectra. Zhu et al. subsequently introduced transfer learning into hyperspectral soybean seed identification and further improved efficiency and accuracy [6], but the method still primarily relied on image-level deep representations instead of directly exploiting the sequential semantics of raw spectra. Li et al. then applied a one-dimensional convolutional neural network to soybean seed hyperspectral classification [7], showing that the raw spectral sequence itself contains substantial discriminative potential; nevertheless, 1D-CNN is mainly effective at extracting local spectral-shape patterns, while its ability to capture long-range wavelength dependency and implicit inter-band relations remains limited. Wei et al. employed ensemble machine learning algorithms for soybean seed hyperspectral classification and improved classification robustness [36], but traditional machine learning approaches still struggle to jointly handle complex nonlinear relations, global wavelength dependency, and fine-grained class boundaries. In interpreting such fine-grained classification, moisture- and morphology-related variability should also be considered. Chang et al. acquired hyperspectral images of soybean grains in the 900–1700 nm range and developed chemometric models for moisture-content estimation [37]. Since both the spectral branch and the GADF-based structural branch are derived from seed-region reflectance spectra, cultivar classification in GMFNet is characterized by multi-wavelength response patterns and inter-band relational information. This provides a more integrated interpretation of the classification process than a single physicochemical or morphological attribute. Compared with these studies, the present work does not rely solely on feature extraction from hyperspectral images, nor does it remain limited to single-branch modeling of raw one-dimensional spectral sequences. Instead, it places raw spectral sequential modeling and relational structural modeling within the same framework.

From the perspective of raw spectral modeling, the superior performance of Mamba in the present study is consistent with the mechanism of the architecture itself. Gu and Dao proposed Mamba as a selective state space model that enables linear-complexity long-sequence modeling, allowing the network to process long-range dependencies more efficiently than conventional recurrent or standard attention-based architectures [25]. Li et al. later introduced MambaHSI for hyperspectral image classification and further demonstrated the potential of state space models for hyperspectral representation learning, especially in capturing long-range interactions across spectral or spatial dimensions [38]. However, the target task of MambaHSI remains conventional hyperspectral image classification in remote sensing, where the emphasis is on spectral–spatial modeling rather than on discriminating one-dimensional seed spectra from individual kernels. In the soybean cultivar classification scenario addressed here, discriminative cues are not concentrated in a few isolated wavelengths, but are distributed across multiple absorption regions, transition intervals, and coordinated changes in the overall spectral profile. Therefore, compared with LSTM, Transformer, or simple convolution–sequence hybrids, Mamba is better suited to preserve wavelength order while simultaneously modeling continuous spectral evolution and long-range inter-band dependency. This explains why it emerges as the strongest backbone among the raw spectral models, while also indicating that raw spectral sequential modeling alone is still insufficient to explicitly reveal latent inter-band relational structure.

From the perspective of two-dimensional structural representation, the introduction of GADF in this study is not intended merely to convert a sequence into an image, but rather to explicitly unfold the latent differential relations among spectral bands. Wang and Oates proposed the GASF/GADF representation in their time-series imaging framework and showed that angular encoding and relational matrix construction can transform one-dimensional sequences into two-dimensional relational patterns that are more amenable to representation learning [39]. This step is particularly meaningful for soybean seed hyperspectral classification because different cultivars may share highly similar global spectral shapes, whereas the relative variation patterns, transition relations, and coordinated differences among local wavelength intervals may constitute the truly discriminative cues. Li et al. proposed a multilevel fusion strategy based on spectral and image information for soybean seed variety identification in 2024 and demonstrated the benefit of multilevel information integration [2]; however, the main focus of their study was the organization of spectral and image information, whereas explicit sequence-to-relational structural conversion was not the central design objective. By contrast, the present work directly constructs GADF from the raw one-dimensional spectrum, so that the two-dimensional branch no longer learns generic image appearance alone, but instead extracts features from an explicit inter-band relational structure. Moreover, the fact that ResNet18 achieved the best generalization performance among all GADF-based models suggests that, for the current GADF data, stable hierarchical structural modeling is more beneficial than simply adopting more complex visual backbones. More importantly, Weighted fusion outperformed Contact fusion, indicating that spectral sequential representation and GADF structural representation are not simply redundant, but strongly complementary.

Although the present study achieved promising classification performance, several aspects still require further improvement. First, the experiments were conducted using a single hyperspectral imaging system under controlled conditions, and the robustness of the model under cross-batch, cross-device, and more complex practical scenarios remains to be further validated. Second, although GMFNet jointly optimizes spectral sequential semantics and GADF-based structural semantics in an end-to-end manner, the current framework still relies mainly on a single spectral source and does not yet incorporate cross-instrument calibration, domain adaptation, or richer phenotypic descriptors, which may limit its transferability in broader application settings. Third, the confusion matrix still reveals local confusion among a few categories, indicating that fine-grained discrimination remains challenging for cultivars with highly similar spectral–structural patterns. Liu et al. emphasized in their review of digital seed phenotyping that future seed analysis will increasingly move toward multisource collaboration, automated integration, and more intelligent interpretation [3]. Accordingly, future work may focus on external validation datasets, robustness assessment across instruments and acquisition batches, wavelength contribution interpretation, and the development of more lightweight and more interpretable fusion strategies to further improve the stability, transferability, and practical applicability of the proposed framework.

## 5. Conclusions

This study developed an end-to-end classification framework, termed GMFNet, for single-seed soybean hyperspectral classification by integrating spectral sequential representation with GADF-based structural representation. Within this framework, the raw reflectance spectral sequence was used to preserve continuous wavelength dependency and global spectral evolution, whereas the GADF-based two-dimensional relational image was introduced to explicitly characterize inter-band differential structure. The two heterogeneous representations were then collaboratively utilized through weighted feature fusion. Experimental results showed that, among the raw spectral models, Mamba achieved the best single-branch sequential modeling performance, while ResNet18 exhibited the strongest generalization ability among the GADF-based structural models. More importantly, the proposed Weighted fusion strategy achieved the best overall performance, reaching accuracies of 0.9039 and 0.9011 on the validation and test sets, respectively, outperforming spectral modeling alone, GADF-based structural modeling alone, and simple concatenation-based fusion.

These findings indicate that spectral sequential semantics and GADF-based structural semantics are highly complementary for fine-grained soybean seed classification. Although each representation provides a certain level of discriminative capability, their full classification potential can only be more effectively realized when they are jointly organized and optimized within a unified framework. Overall, this study provides an effective fusion-based modeling paradigm for single-seed soybean hyperspectral classification and offers a useful reference for combining one-dimensional spectral representation with two-dimensional relational representation in fine-grained agricultural hyperspectral recognition. Beyond offline model evaluation, translating this framework into on-site soybean seed inspection requires stable hyperspectral acquisition, controlled illumination, standardized single-seed conveying and presentation, reliable background suppression and seed-region segmentation, real-time preprocessing and inference, and calibration transfer across instruments, batches, and environments. Integration with automated conveying, online sorting, and quality-control modules is also necessary to establish an operational single-seed inspection workflow. Future work may further focus on cross-device and cross-batch validation, external dataset evaluation, lightweight model design, and interpretable feature analysis to improve the robustness and practical applicability of the proposed framework.

## Figures and Tables

**Table 1:** Class labels, cultivar names, and sample distributions in the training, validation, and test sets.

Class Label	Cultivar	Total Samples	Training Set	Validation Set	Test Set
0	Rudong Popi Feng	3100	2066	517	517
1	Hanjiang Hongdadou	3100	2067	517	516
2	Liyang Chadou Bayuehuang	3100	2067	516	517
3	Taicang Ziyoudou	3100	2067	517	516
4	Dantu Huangdou	3100	2066	517	517
5	Nantong Yuandou	3100	2067	516	517
6	Yangzhou Wuyuedou	3100	2067	516	517
7	Wujin Xizi	3100	2066	517	517

**Table 2:** Classification performance of models based on spectral representation.

Models	Train	Val	Test	Recall	F1-Score
SVC	0.8143	0.8151	0.8209	0.8207	0.8188
LSTM	0.8280	0.8251	0.8215	0.8212	0.8240
1D-CNN	0.8662	0.8614	0.8570	0.8568	0.8586
Transformer	0.8562	0.8485	0.8491	0.8489	0.8469
Mamba	**0.8906**	**0.8808**	**0.8721**	**0.8719**	**0.8748**
CNN-LSTM	0.9080	0.8628	0.8662	0.8660	0.8671
CNN-Transformer	0.8374	0.8314	0.8210	0.8208	0.8227

The bolded data shows the best results for all models in the table.

**Table 3:** Classification performance summary of spectral models under three random-seed data partitions.

Models	Test	Recall	F1-Score
SVC	0.8205 ± 0.0008	0.8203 ± 0.0008	0.8193 ± 0.0008
LSTM	0.8226 ± 0.0019	0.8223 ± 0.0019	0.8229 ± 0.0018
1D-CNN	0.8577 ± 0.0013	0.8575 ± 0.0013	0.8578 ± 0.0013
Transformer	0.8486 ± 0.0009	0.8484 ± 0.0009	0.8474 ± 0.0009
Mamba	**0.8732 ± 0.0019**	**0.8730 ± 0.0019**	**0.8737 ± 0.0019**
CNN-LSTM	0.8667 ± 0.0009	0.8665 ± 0.0009	0.8665 ± 0.0010
CNN-Transformer	0.8219 ± 0.0015	0.8217 ± 0.0015	0.8219 ± 0.0014

The bolded data shows the best results for all models in the table.

**Table 4:** Classification performance of GADF-based structural models and fusion strategies under one random-seed data partitions.

	Models	Train	Val	Test	Recall	F1-Score
GADF	2D-CNN	0.8405	0.8439	0.8498	0.8495	0.8491
	ResNet18	**0.8953**	**0.8668**	**0.8737**	**0.8735**	**0.8730**
	Swin-Tiny	0.9118	0.8587	0.8505	0.8503	0.8498
	Visual Mamba	0.9102	0.8488	0.8500	0.8498	0.8493
	FasterViT	0.8771	0.8523	0.8655	0.8653	0.8648
	ConvNeXt V2	0.9238	0.8546	0.8556	0.8554	0.8549
Fusion	Contact	0.9068	0.8831	0.8895	0.8893	0.8889
	Weighted	**0.9618**	**0.9039**	**0.9011**	**0.9009**	**0.9004**

Bold values indicate the best-performing model within each feature representation group.

**Table 5:** Classification performance summary of GADF-based structural models and fusion strategies under three random-seed data partitions.

	Models	Test	Recall	F1-Score
GADF	2D-CNN	0.8486 ± 0.0013	0.8483 ± 0.0013	0.8479 ± 0.0013
	ResNet18	**0.8738 ± 0.0023**	**0.8736 ± 0.0024**	**0.8731 ± 0.0024**
	Swin-Tiny	0.8507 ± 0.0020	0.8505 ± 0.0020	0.8500 ± 0.0020
	Visual Mamba	0.8502 ± 0.0019	0.8500 ± 0.0019	0.8495 ± 0.0018
	FasterViT	0.8651 ± 0.0020	0.8649 ± 0.0020	0.8644 ± 0.0020
	ConvNeXt V2	0.8559 ± 0.0022	0.8557 ± 0.0022	0.8552 ± 0.0022
Fusion	Contact	0.8895 ± 0.0023	0.8893 ± 0.0023	0.8889 ± 0.0024
	Weighted	**0.9014 ± 0.0025**	**0.9012 ± 0.0025**	**0.9008 ± 0.0024**

Bold values indicate the best-performing model within each feature representation group.

## Data Availability

The original contributions presented in this study are included in the article. Further inquiries can be directed to the corresponding authors.
